# Identifying Patients Who Are Likely to Receive Most of Their Care From a Specific Health Care System: Demonstration via Secondary Analysis

**DOI:** 10.2196/12241

**Published:** 2018-11-05

**Authors:** Gang Luo, Peter Tarczy-Hornoch, Adam B Wilcox, E Sally Lee

**Affiliations:** 1 Department of Biomedical Informatics and Medical Education University of Washington Seattle, WA United States; 2 Division of Neonatology Department of Pediatrics University of Washington Seattle, WA United States; 3 Department of Computer Science and Engineering University of Washington Seattle, WA United States; 4 Population Health Analytics University of Washington Medicine Finance University of Washington Seattle, WA United States

**Keywords:** data analysis, inpatients, emergency departments, health care system

## Abstract

**Background:**

In the United States, health care is fragmented in numerous distinct health care systems including private, public, and federal organizations like private physician groups and academic medical centers. Many patients have their complete medical data scattered across these several health care systems, with no particular system having complete data on any of them. Several major data analysis tasks such as predictive modeling using historical data are considered impractical on incomplete data.

**Objective:**

Our objective was to find a way to enable these analysis tasks for a health care system with incomplete data on many of its patients.

**Methods:**

This study presents, to the best of our knowledge, the first method to use a geographic constraint to identify a reasonably large subset of patients who tend to receive most of their care from a given health care system. A data analysis task needing relatively complete data can be conducted on this subset of patients. We demonstrated our method using data from the University of Washington Medicine (UWM) and PreManage data covering the use of all hospitals in Washington State. We compared 10 candidate constraints to optimize the solution.

**Results:**

For UWM, the best constraint is that the patient has a UWM primary care physician and lives within 5 miles of at least one UWM hospital. About 16.01% (55,707/348,054) of UWM patients satisfied this constraint. Around 69.38% (10,501/15,135) of their inpatient stays and emergency department visits occurred within UWM in the following 6 months, more than double the corresponding percentage for all UWM patients.

**Conclusions:**

Our method can identify a reasonably large subset of patients who tend to receive most of their care from UWM. This enables several major analysis tasks on incomplete medical data that were previously deemed infeasible.

## Introduction

In the United States, health care is fragmented in numerous distinct health care systems including private, public, and federal organizations like private physician groups and academic medical centers. Frequently, a given health care system has incomplete medical data on many of its patients, as these patients’ complete data are recorded across multiple health care systems [1,2]. Finnell et al [2] showed that during a 3-year period in Indiana, 40.7% of emergency department visits came from patients who also had emergency department visits at other health care systems. Bourgeois et al [1] showed that during a 5-year period in Massachusetts, 56.5% of adult hospital encounters (inpatient stays and emergency department visits) came from patients who also had encounters at other hospitals. Incomplete data are particularly problematic in academic health care systems such as the University of Washington Medicine (UWM), where many patients are referred from other health care systems. As shown in the Results section, less than one-third of the hospital encounters for all UWM patients occur within UWM. Currently, several major data analysis tasks such as predictive modeling using historical data are deemed impractical on incomplete data. This limits the applications based on these analysis tasks. For example, predictive modeling is widely used for identifying future high-cost patients [3] for care management [4] to prevent high costs and health status degradation [5-7]. Typical models for projecting a patient’s cost assume complete historical data [8-10] and are not used by a health care system with incomplete data on its patients. As a result, many future high-cost patients are not identified and enrolled in care management, contributing to undesirable outcomes.

This study presents, to the best of our knowledge, the first method to use a geographic constraint to identify a reasonably large subset of patients who tend to receive most of their care from a specific health care system. This is to enable these data analysis tasks on incomplete medical data. Although the health care system has incomplete data on many of its patients, it has more complete data on this subset of patients. For a data analysis task requiring relatively complete medical data, we can conduct the task for this subset of patients, with the understanding that the analysis results apply to only this subset of patients rather than all patients of the health care system. This could be an improvement compared with the current practice of not conducting the task at all, in cases when conducting the task on all patients is impractical. Our previous work [11] sketched the method’s main goal but did not complete the method, do a computer coding implementation, or evaluate the method’s performance. This study aims to fill these gaps and demonstrate our method using data at UWM.

## Methods

### Patient Population

The patient cohort included all adult patients (aged ≥18 years) who had encounters at UWM facilities (hospitals and clinics) with information stored in UWM’s enterprise data warehouse during the 1-year period of April 1, 2016, to March 31, 2017. In this paper, an encounter can be of any type, unless it is explicitly specified as a hospital encounter or an outpatient visit. UWM is the largest academic health care system in Washington State and has both hospitals and clinics for adults.

### Dataset

We used administrative data in UWM’s enterprise data warehouse during the 2-year period of April 1, 2015, to March 31, 2017. The dataset included encounter and primary care physician (PCP) information of our patient cohort. We also used PreManage data that UWM has on all of its patients during the 6-month period of April 1, 2017, to September 30, 2017. PreManage is Collective Medical Technologies Inc’s commercial product offering encounter and diagnosis data on hospital encounters (inpatient stays and emergency department visits) at many US hospitals [12]. PreManage data cover all hospitals in Washington State. Starting April 1, 2017, UWM has been receiving relatively complete PreManage data on its patients. In this paper, we chose April 1, 2017, as the index date separating the prior and subsequent periods for the analysis task.

### Our Constraint-Based Patient Identification Method

Our goal is to use a constraint to identify a reasonably large subset of patients who tend to receive most of their care from UWM. We considered 3 UWM hospitals whose administrative and clinical data are stored in the UWM’s enterprise data warehouse: Harborview Medical Center, University of Washington Medical Center, and Northwest Hospital. All the 3 hospitals are in Seattle, Washington. We considered the following candidate constraints that all include the component of living within *r* miles of at least one of the 3 UWM hospitals, with *r* being a parameter whose optimal value was to be determined in the study:

1. Distance only: The patient lives within *r* miles of at least one of the 3 UWM hospitals. Intuitively, with everything else being equal, the closer a patient lives to UWM hospitals, the larger portion of the patient’s care tends to be received from UWM. In addition, the smaller the *r*, the smaller the number of UWM patients satisfying the constraint.
2. PCP: The patient has a UWM PCP and lives within *r* miles of at least one of the 3 UWM hospitals. UWM PCPs tend to make referrals within UWM. Hence, intuitively, compared with others, patients with a UWM PCP may receive a larger portion of their care from UWM.
3. ≥2 encounters in the past year: The patient had ≥2 encounters at UWM facilities in the past year (April 1, 2016, to March 31, 2017) and lives within *r* miles of at least one of the 3 UWM hospitals. Intuitively, patients with more previous encounters at UWM facilities may receive a larger portion of their care from UWM in the future.
4. ≥2 encounters in the past 2 years: The patient had ≥2 encounters at UWM facilities in the past 2 years (April 1, 2015, to March 31, 2017) and lives within *r* miles of at least one of the 3 UWM hospitals.
5. ≥1 hospital encounter in the past year: The patient had ≥1 hospital encounter in 1 of the 3 UWM hospitals in the past year and lives within *r* miles of at least one of the 3 UWM hospitals.
6. ≥2 hospital encounters in the past year: The patient had ≥2 hospital encounters across the 3 UWM hospitals in the past year and lives within *r* miles of at least 1 of the 3 UWM hospitals.
7. ≥2 hospital encounters in the past 2 years: The patient had ≥2 hospital encounters across the 3 UWM hospitals in the past 2 years and lives within *r* miles of at least one of the 3 UWM hospitals.
8. ≥1 outpatient visit in the past year: The patient had ≥1 outpatient visit to UWM in the past year and lives within *r* miles of at least one of the 3 UWM hospitals.
9. ≥2 outpatient visits in the past year: The patient had ≥2 outpatient visits to UWM in the past year and lives within *r* miles of at least one of the 3 UWM hospitals.
10. ≥2 outpatient visits in the past 2 years: The patient had ≥2 outpatient visits to UWM in the past 2 years and lives within *r* miles of at least one of the 3 UWM hospitals.

In each candidate constraint, distance is no longer a factor when *r*=+∞.

### Data Analysis

Using the distVincentyEllipsoid function in R’s geosphere package version 1.5-5 [13], we computed the ellipsoid great circle distance between a patient’s home and a UWM hospital based on the longitude and latitude coordinates of the patient’s 5-digit home address zip code and the hospital’s address. This distance serves as a rough proxy of the travel distance between the patient’s home and hospital, is easy to compute, and is sufficient for our patient identification purpose, as shown in the Results section. For other researchers wanting to adopt our constraint-based patient identification method for their studies, using zip codes instead of exact patient home addresses can facilitate data acquisition because a limited dataset is easier to obtain than an identified one.

We compared performance across the 10 candidate constraints for identifying patients likely to receive most of their care from UWM. We used administrative data in UWM’s enterprise data warehouse to check whether a patient satisfied a specific constraint. For each candidate constraint, we computed the percentage of UWM patients satisfying it. For all patients satisfying the constraint, we used PreManage data to compute the percentage of their hospital encounters that occurred within UWM in the following 6 months (April 1, 2017, to September 30, 2017). As hospital encounters are usually much more expensive than other encounters, this percentage reflects the portion of these patients’ care received from UWM. In computing this percentage, every patient satisfying the constraint was included, regardless of whether the patient had ≥1 hospital encounter in the following 6 months. When selecting the final constraint to be used, we struck a balance between the following 2 criteria:

Criterion 1: The percentage of UWM patients satisfying the constraint should be as large as possible. As multiple data analysis tasks will be conducted on these patients, this will maximize the usefulness of the applications based on these tasks.

Criterion 2: For the patients satisfying the constraint, the percentage of their hospital encounters that occurred within UWM should be as large as possible. This is to maximize the degree of completeness of the medical data that UWM has on these patients. For the data analysis tasks that will be conducted on these patients, this degree impacts the biases in the analysis results.

As mentioned in the Discussion section, the selected constraint has a special property, increasing our confidence that the patients identified by the constraint also tend to incur most of their outpatient visits within UWM.

To show how the constraint-based method works for individual UWM hospitals, for all patients satisfying the selected constraint and each of the 3 UWM hospitals, we used PreManage data to compute the percentage of these patients’ hospital encounters that occurred at the UWM hospital in the following 6 months.

### Ethics Approval

The institutional review board of UWM reviewed and approved this study and waived the need for informed consent for all patients.

## Results

Table 1 shows the demographic characteristics of our patient cohort.

**Figure 1:**
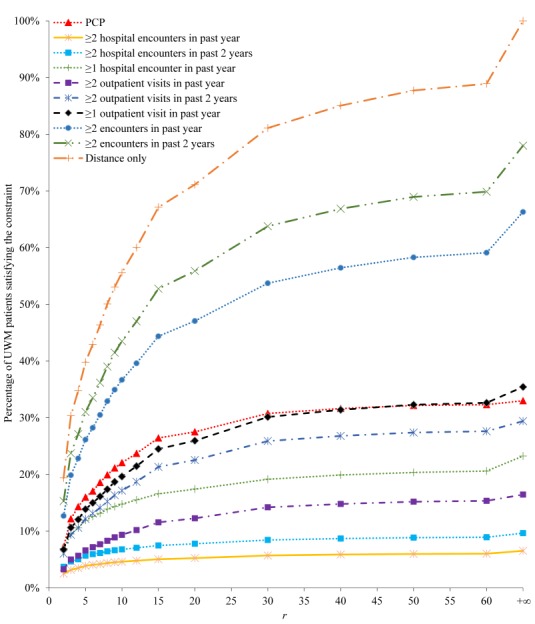
The percentage of University of Washington Medicine (UWM) patients satisfying each of the 10 candidate constraints. PCP: primary care physician.

**Figure 2:**
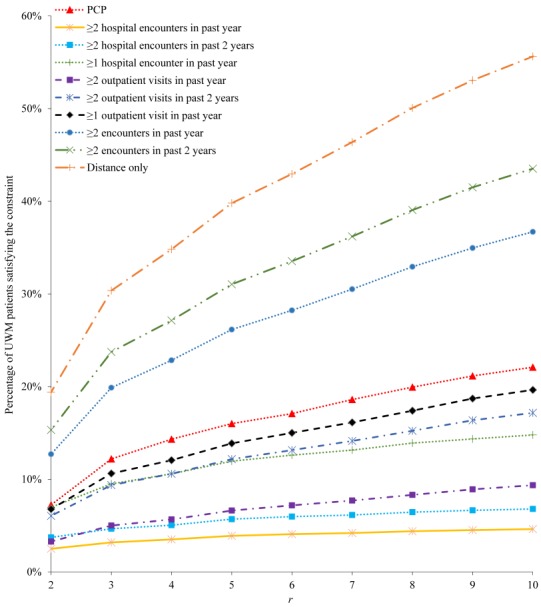
The percentage of University of Washington Medicine (UWM) patients satisfying each of the 10 candidate constraints, when r is ≤10. PCP: primary care physician.

Figures 1 and 2 show the percentage of UWM patients satisfying each of the 10 candidate constraints. The percentage increases with *r*, initially quickly when *r* is small and then more slowly as *r* becomes larger. Recall that *r* is the maximum allowed distance in miles between the patient’s home and the closest UWM hospital. As UWM mainly serves the Seattle metropolitan area, 88.92% (309,483/348,054) of UWM patients live within 60 miles of at least one of the 3 UWM hospitals. About 44.76% (138,530/309,483) of these patients live within 5 miles.

**Figure 3:**
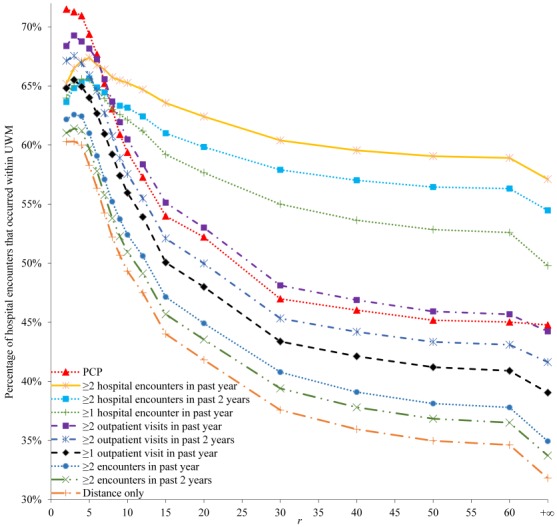
The percentage of hospital encounters that occurred within the University of Washington Medicine (UWM) in the following 6 months for each of the 10 candidate constraints and all patients satisfying it. PCP: primary care physician.

**Figure 4:**
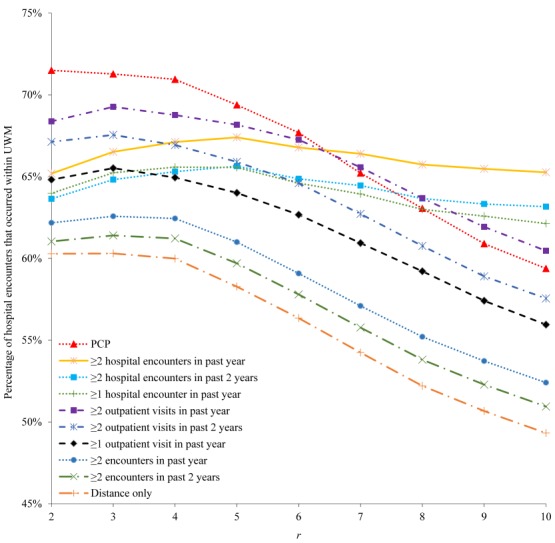
The percentage of hospital encounters that occurred within the University of Washington Medicine (UWM) in the following 6 months for each of the 10 candidate constraints and all patients satisfying it, when r is ≤10. PCP: primary care physician.

For each of the 10 candidate constraints and all the patients satisfying it, Figures 3 and 4 show the percentage of their hospital encounters that occurred within UWM in the following 6 months. With a few exceptions when *r* is small, as *r* increases, the percentage decreases, initially quickly when *r* is small and then more slowly as *r* becomes larger. This is consistent with our intuition that with everything else being equal, patients living further from UWM hospitals are less likely to use them. Regardless of how small *r* is, this percentage never approaches 100%, partly because UWM patients could also use several non-UWM hospitals that are within 1 mile of certain UWM hospitals. As we want this percentage to be as large as possible, we should choose *r* to be ≤5 or ≤10, depending on the constraint.

**Table 1:** Demographic characteristics of adult patients who had encounters at the University of Washington Medicine (UWM) facilities with information stored in UWM’s enterprise data warehouse during April 1, 2016, to March 31, 2017 (N=348,054).

Demographic characteristics			n (%)
**Age in years**			
	18 to <30	64,311 (18.48)	
	30 to <65	209,033 (60.06)	
	≥65	74,710 (21.47)	
**Gender**			
	Male	154,511 (44.39)	
	Female	193,506 (55.60)	
	Unknown or not reported	37 (0.01)	
**Race**			
	American Indian or Alaska native	5107 (1.47)	
	Asian	33,595 (9.65)	
	Black or African American	25,224 (7.25)	
	Multiple races	2772 (0.80)	
	Native Hawaiian or other Pacific islander	2500 (0.72)	
	White	232,354 (66.76)	
	Unknown or not reported	46,502 (13.36)	
**Ethnicity**			
	Hispanic	21,436 (6.16)	
	Non-Hispanic	270,043 (77.59)	
	Unknown or not reported	56,575 (16.25)	
**Insurance**			
	Private	167,640 (48.16)	
	Public	154,008 (44.25)	
	Self-paid or charity	26,406 (7.59)	

**Figure 5:**
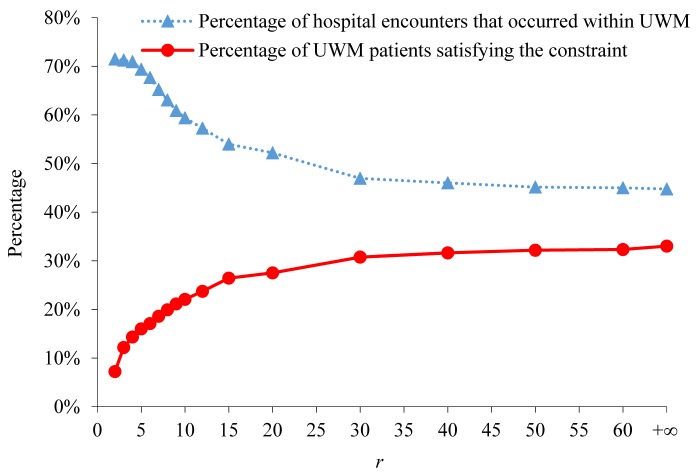
The percentage of University of Washington Medicine (UWM) patients satisfying the PCP constraint and the percentage of these patients’ hospital encounters that occurred within UWM in the following 6 months. PCP: primary care physician.

In selecting the final constraint to be used, we struck a balance between Criteria 1 and 2 listed at the end of the Methods section. The *PCP* constraint significantly outperforms 6 other constraints on Criterion 1. When *r* is ≤6, the *PCP* constraint outperforms all the other constraints under Criterion 2. Also, when *r*=+∞ and distance is no longer a factor, no constraint outperforms the *PCP* constraint with *r* ≤6 under Criterion 2. Figure 5 shows the percentage of UWM patients satisfying the *PCP* constraint as well as the percentage of these patients’ hospital encounters that occurred within UWM in the following 6 months. When the *PCP* constraint was used with *r*=5, 16.01% (55,707/348,054) of UWM patients satisfied the constraint. For these patients, 69.38% (10,501/15,135) of their hospital encounters occurred within UWM in the following 6 months. In comparison, for all UWM patients, 31.80% (39,171/123,162) of their hospital encounters occurred within UWM in the following 6 months.

**Figure 6:**
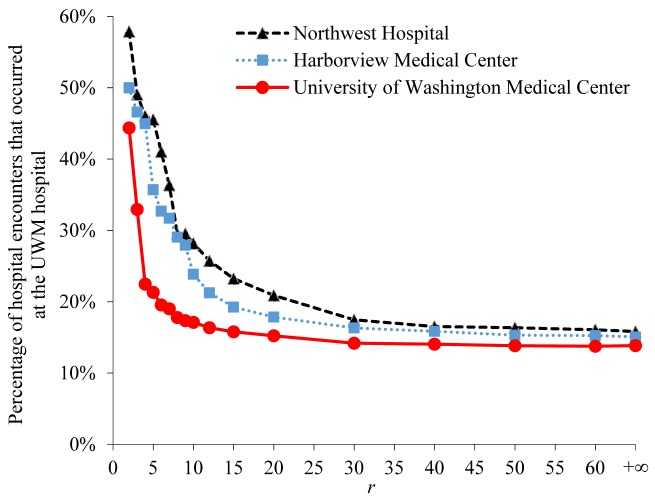
For each of the 3 University of Washington Medicine (UWM) hospitals and all patients satisfying the PCP constraint, the percentage of their hospital encounters that occurred at the UWM hospital in the following 6 months. PCP: primary care physician.

For each of the 3 UWM hospitals and all patients satisfying the *PCP* constraint, Figure 6 shows the percentage of their hospital encounters that occurred at the UWM hospital in the following 6 months. The percentage varies across the 3 UWM hospitals. As *r* increases, the percentage decreases at similar rates across the 3 UWM hospitals.

## Discussion

### Principal Findings

By striking a balance between Criteria 1 and 2, we chose the *PCP* constraint with *r*=5 as the final one to be used. Using our constraint-based method to identify the right subset of patients, we more than doubled the percentage of patient hospital encounters that occurred within UWM in the following 6 months: from 31.80% (39,171/123,162) to 69.38% (10,501/15,135). Moreover, as each identified patient has a UWM PCP, we are confident that the identified patients incurred most of their outpatient visits within UWM in the following 6 months, even if we do not have data to verify this.

### Potential Use of Our Results

Our results show that for patients living within 5 miles of at least one of the 3 UWM hospitals, UWM provides most of their care and has reasonably complete medical data on them. For a data analysis task requiring relatively complete data, such as predictive modeling using historical data, we can conduct the task on this subset of patients and obtain useful results, even if conducting the task on all UWM patients is impractical. For example, we can build a predictive model to identify future high-cost patients among this subset [3]. Enrolling such patients in care management can help prevent high costs and improve outcomes [5-7].

Our results show that patients living further from the 3 UWM hospitals tend to receive a smaller portion of their care from UWM. This suggests UWM to consider using different preventive interventions for patients living at differing distances from the UWM hospitals, for example, for care management to achieve better results. For patients who will receive only a small portion of their care from UWM, it is difficult for UWM to use expensive preventive interventions in a cost-effective manner.

This study used PreManage data covering adult patients in all age groups. It cannot be done using Medicare claims data that mainly cover patients aged ≥65 years and patients with certain disabilities and diseases. Similar to many other health care systems, UWM does not have complete claims data covering all its patients’ health care use both within and outside of UWM. We could use claims data to do a similar study for another health care system if it has complete claims data covering all its patients’ health care use both within and outside of that system.

This study used PreManage data to validate the *PCP* constraint’s effectiveness for UWM. However, the *PCP* constraint does not depend on PreManage data’s availability and can be used by another health care system even if it cannot access PreManage data. In this case, one way to estimate the *PCP* constraint’s effectiveness is to survey some of its patients about their health care use both within and outside of the system.

This paper focuses on identifying patients likely to receive most of their care from a specific health care system. If multiple health care systems exchange data, we could use a similar method to identify patients likely to receive most of their care from these health care systems combined. This could enable several data analysis tasks across these health care systems.

### Limitations

This study has several limitations that can serve as interesting areas for future work:

- So far, UWM has accumulated PreManage data only over a limited period. After UWM accumulates more PreManage data, we should redo our analysis, check the percentage of patient hospital encounters that occur within UWM in the next 2 to 3 years, and see whether any of our conclusions will change.
- This study demonstrates our constraint-based patient identification method at a single health care system, UWM, which provides both inpatient and outpatient care mainly for an urban area. To understand how our method generalizes, we should repeat our analysis on several other health care systems, some mainly serving urban areas and others offering many services in rural areas, and see whether the optimal constraint will change. For a health care system offering many services in rural areas, we would expect the optimal value of *r* to be >5, as patients are more scattered in rural areas than in urban areas.
- For a health care system with incomplete medical data on many of its patients, we can use our method to identify a subset of patients on whom the health care system has more complete data and estimate the data’s incompleteness level on this subset of patients. For a data analysis task, using incomplete data to do the analysis on this subset of patients would produce biased results, which could still be better than no result if the degree of bias is acceptable. Yet, the exact relationship between data incompleteness level and degree of bias in the analysis results is unknown. In particular, we have no idea of the threshold for the data’s incompleteness level, beyond which the analysis’ conclusion could become invalid. To address this issue, we can take a reasonably complete dataset from another health care system such as Kaiser Permanente, remove different portions of the dataset, and check the resulting impact on the analysis results. This will help us understand whether our method is good enough for enabling the data analysis task in the current health care system.

### Conclusions

To the best of our knowledge, for a health care system with incomplete medical data on many of its patients, we provided the first method to use a geographic constraint to identify a reasonably large subset of patients who tend to receive most of their care from the system. Our results show that our method performs reasonably well at UWM. Our method opens the door for conducting several major analysis tasks on incomplete medical data, which were previously deemed impractical to undertake.

## Abbreviations

PCP: primary care physician
UWM: University of Washington Medicine
